# Pridopidine Mediated Sigma-1 Receptor Activation and Therapeutic Implications in Neurodegenerative Diseases

**DOI:** 10.3390/neurolint18080144

**Published:** 2026-07-29

**Authors:** Ahmed I. Anwar, Abdul-rahman A. Hegazi, Hamsa Priya Bhuchakra, Joshua R. Nelson, Ty L. Birdsong, Cy J. Fontenot, Majed Zeibo, Moiz M. Fazal-ur-Rehman, Harrison P. Bieber, Claudia J. Spring, Joshua L. Smith, Ibraheem A. Hachem, Taranjit Singh, M. Farris Sawaya, Kevin S. Murnane, Alan D. Kaye

**Affiliations:** 1 School of Medicine, Louisiana State University Health Shreveport, Shreveport, LA 71103, USA; 2 Department of Anesthesiology, Louisiana State University Health Shreveport, Shreveport, LA 71103, USA; 3 Department of Pharmacology, Toxicology & Neuroscience, School of Graduate Studies, Louisiana State University Health Shreveport, Shreveport, LA 71103, USA; 4 Louisiana Addiction Research Center, Louisiana State University Health Shreveport, Shreveport, LA 71103, USA; 5 Department of Psychiatry and Behavioral Medicine, School of Medicine, Louisiana State University Health Shreveport, Shreveport, LA 71103, USA

**Keywords:** sigma-1-receptor, S1R, pridopidine, neurodegeneration, Huntington’s disease, amyotrophic lateral sclerosis

## Abstract

Neurodegenerative diseases are targets for pridopidine therapy, which aims to improve quality of life through neuroprotective mechanisms that involve sigma-1 receptor (S1R) activation. Neurodegenerative motor and cognitive diseases are influenced by dopamine imbalance, where disruptions in pathways contribute to states that are hyperkinetic or hypokinetic, while current dopaminergic treatments are symptomatic rather than disease-modifying, especially for Huntington’s disease and Amyotrophic lateral sclerosis. This review summarizes the mechanisms underlying pridopidine-mediated neuroprotection and examines the current evidence supporting its therapeutic potential. The S1R is an endoplasmic reticulum-mitochondria-associated chaperone involved in homeostasis of calcium, stress regulation, and mitochondrial function. Pridopidine is a small lipophilic molecule that crosses the blood–brain barrier and acts as an S1R agonist, with minimal dopamine D2 receptor occupancy. Activation of S1R by pridopidine modulates calcium signaling and enhances anti-apoptotic activity. Collectively, available evidence suggests that pridopidine may improve motor outcomes and slow disease progression in Huntington’s disease and amyotrophic lateral sclerosis, supporting its promise as a disease-modifying therapeutic strategy.

## 1. Introduction

Neurodegenerative diseases have become an increasingly popular target for new pharmacological agents such as pridopidine. The role of pridopidine in the treatment of neurodegenerative diseases, such as Huntington’s disease (HD), amyotrophic lateral sclerosis (ALS), and related disorders, is to specifically target sigma-1 receptors (S1R) and provide neuroprotection [1]. HD is an autosomal dominant disorder caused by a trinucleotide repeat expansion of CAG in the *HTT* gene on chromosome 4, affecting the huntingtin protein. HD can lead to both motor and cognitive dysfunction that can hinder daily life, and most patients have a median survival of 15–20 years after clinical symptoms begin [2].

ALS is also a neurodegenerative disease caused by upper and lower motor neuron degeneration, leading to asymmetric muscle weakness and eventually death, commonly caused by respiratory failure [3]. The median survival of ALS is 2–4 years after diagnosis, and long-term mechanical ventilation can prolong death and improve the quality of life for patients [4]. Other related disorders include Parkinson’s disease, Wolfram syndrome, and Rett syndrome, all characterized by progressive neurodegeneration.

In HD, functional decline is a concern as total functional capacity (TFC) measures patients’ ability to live and function every day, and HD is currently being treated with VMAT2 inhibitors, tetrabenazine, and deutetrabenazine, to address chorea, a movement disorder that involves sudden, irregular movements of the limbs, neck, head, and/or face.

Antipsychotics and antidepressants are also used in HD to treat behavioral symptoms that appear in HD, such as irritability, psychosis, and depression. ALS treatment focuses on extending survival due to low survival rates following diagnosis, and therapy includes Riluzole, a sodium channel inhibitor that reduces glutamate release and reduces excitotoxicity in the central nervous system (CNS), and Edaravone, a reactive oxygen species (ROS) scavenger [5]. The treatments available for HD and ALS face the difficulties of finding clinical improvements related to disease processes of heterogeneity and late intervention windows [6].

Dopamine signaling requires a balance to maintain motor and cognitive function through direct and indirect pathways in the CNS. Motor symptoms of neurodegenerative diseases involve both hyperkinetic and akinetic stages related to the imbalance of dopamine [7]. Indirect pathway degeneration can lead to hyperkinesia, as increased dopamine suppresses the indirect pathway’s inhibitory effects via D2 receptors. In contrast, direct pathway degeneration can produce bradykinesia and/or akinesia, as later dopamine depletion can inhibit excitatory effects via D1 receptor damage [8]. Cognitive dysfunction can result from D1 and D2 receptor loss, leading to decreased behavioral flexibility, working memory, and executive function [9]. Dopaminergic drugs that play a role in neurodegenerative disease treatment, like VMAT2 inhibitors and dopamine antagonists, can be used for chorea by decreasing the amount of dopamine signaling; however, these drugs do not have any effect on modifying the disease [10].

S1R is a cell-surface receptor used to treat neurodegenerative disorders. S1R is a chaperone protein in the endoplasmic reticulum-mitochondria-associated membrane (MAM) that regulates calcium homeostasis, endoplasmic reticulum (ER) stress response, mitochondrial function, proteostasis, and neuroinflammation [11]. In response to ER stress and proteostasis challenges, S1R regulates the unfolded protein response (UPR), which is activated when misfolded proteins build up in the ER. S1R binds and stabilizes the transmembrane receptors of the ER to prevent degradation and repair misfolded proteins [12]. S1R can also enable protein clearance by binding RNA and enhancing autophagosome formation [13]. Regulation of Bcl-2 expression is influenced by S1R, as S1R suppresses intracellular ROS, thereby inhibiting NF-kB and promoting cell survival [14]. Brain-Derived Neurotrophic Factor (BDNF) expression is also activated by S1R activation and contributes to the BDNF axonal transport deficits observed in neurodegenerative diseases [1].

Pridopidine, a selective S1R agonist, has been developed to treat neurodegenerative diseases; however, it was originally thought to target dopamine receptors. It was classified as a “dopamine stabilizer” because it was thought to act mainly at D2 receptors; however, PET images and data showed that only ~3% of D2/D3 receptors were occupied, and over 90% of brain S1Rs were occupied by pridopidine [15]. As a selective S1R agonist, pridopidine modulates calcium homeostasis, the ER stress response, mitochondrial function, proteostasis, and neurotrophic signaling. The selectivity of pridopidine allows it to concentrate its effects at the ER MAM and employ neuroprotective effects on a single receptor target [1]. Pridopidine is a small, lipophilic molecule that readily crosses the blood–brain barrier, showing cooperative binding and over 90% S1R occupancy throughout the brain [15]. The drug has an oral bioavailability close to 100% and is metabolized by CYP2D6 and inhibits CYP2D6 via autoinhibition [16]. With repeat dosing, pridopidine can eventually inactivate the very enzyme that metabolizes it and is renally excreted. The commonly reported side effects include insomnia, diarrhea, nausea, and dizziness with a dose-dependent QT prolongation [17]. Due to its inhibition of CYP2D6, pridopidine should be monitored when used with beta-blockers, SSRIs, and TCAs [18].

The purpose of this study, therefore, is to determine the efficacy of pridopidine in those diagnosed with neurodegenerative diseases. Previous clinical trials of neuroprotection in HD and ALS have not shown a significant difference in TFC; the drug remains under investigation. In HD trials, pridopidine was found to be favored in comparison to patients not receiving antidopaminergic medications [19]. ALS trials did not find a significant difference between pridopidine and placebo at the TFC endpoint [20]. The limitations of previous trials include the disease process of both HD and ALS. The diseases are heterogeneous, making it difficult to provide a specific treatment plan due to patients’ different genetic backgrounds [20]. Biomarkers of pharmacodynamic activity have not been confirmed in previous trials, so although pridopidine shows occupancy, it does not necessarily indicate that the drug is functionally active in these areas [15]. The purpose of this review is to evaluate the efficacy of pridopidine in the treatment of neurodegenerative diseases by examining its mechanisms of action and the available evidence.

## 2. Historical and Clinical Development of Pridopidine

Pridopidine (ACR16), a member of a class of dopamine agonists, was first discovered by the Carlsson research group and was postulated to be a potential treatment for schizophrenia because of stabilizing actions on presynaptic dopamine receptors. These drugs were thought to act as dopamine antagonists at high levels and to stabilize dopamine-mediated actions at subnormal levels [21].

The implication of the dual dopaminergic actions gave ACR16 the potential to prevent the positive symptoms of schizophrenia without the symptoms of dopamine blockade. This theory was supported by subsequent data comparing pridopidine to typical antipsychotics; pridopidine showed striatal D2 antagonism at levels corresponding to elevated in vivo dopamine levels with stimulatory effects in habituated rat models compared to the solely inhibitory effects seen by treatment with haloperidol. These findings, however, did not support direct inhibitory action of pridopidine on the D2 receptors but rather an inhibitory interaction with active forms of the receptor [22,23].

This evidence was further supported by analysis of rat models tested with strong N-methyl-D-aspartate (NMDA) antagonists compared to NMDA agonists after administration of pridopidine. The rats in each group showed retained spontaneous activity reduction in the NMDA antagonist group and a reduction in hyperactivity with preservation of spontaneous motor activity in the NMDA agonist group. These findings supported the dual action on D2 dopamine receptors previously reported [24].

The dual action of pridopidine raised interest for its use in the treatment of other neurodegenerative diseases with dopaminergic involvement. HD became a particular disease of interest due to the dual nature of dopamine involvement, with dopamine being elevated early in the disease and reduced late in the disease process [25]. Early small studies showed promising results with statistically significant improvement in hypokinetic symptoms and an unremarkable safety profile [26]. These results prompted large-scale trials such as the MermaiHD trial and the HART trial. The MermaiHD trial was completed first (2011) and analyzed two doses of pridopidine over 6 months in a double-blinded placebo-controlled trial. The primary result showed a statistically insignificant reduction in the modified motor score (mMS); however, it did show functional improvements in patients such as balance, cutlery usage, and eye movement symptoms [27]. These positively leaning results were supported by similar findings in the HART trial (2013), which showed no statistically significant improvement in the mMS but did show improvement in voluntary motor function [28].

While these trials showed promising results, the unclear mechanism of pridopidine hinders accurate study and analysis of the clinical application of this medication to patients with HD. Further study into the mechanism of action of pridopidine showed binding affinity to S1R in the endoplasmic reticulum [29]. Elevated action of S1R has been associated with neurodegeneration of striatal medium spiny neurons (MSMs) in mice with HD. Treatment with pridopidine reduced the MSM spine loss observed in the control group and restored calcium homeostasis [29]. Proper calcium regulation by S1R modulation seems to show a neuroprotective effect of pridopidine on neurons typically affected by HD.

The S1R-mediated neurodegeneration seems to be a broader mechanism that is potentially displayed in Alzheimer’s disease [30], ALS [31], and frontotemporal dementia [32]. ALS has been further explored so far as a potential disease process that pridopidine could be useful in treating. Early mouse models showed that SOD1 mutated mice showed reduced cachexia after treatment with pridopidine [33]. Neuron progenitor cell survival has also been shown to increase after treatment with pridopidine in stem cell studies. These studies showed a reduction in pro-apoptotic signaling pathway expression as well as the previously mentioned calcium and mitochondrial stabilization [34]. While these results are promising, the HEALEY ALS Platform Trial, a double-blinded placebo-controlled trial, did not show improvement in primary or secondary endpoints, including functional metrics as well as survival, over 24 weeks [35].

Pridopidine is not currently approved for treatment for any disease process across the United States or Europe; however, phase 3 clinical trials are ongoing in the treatment of HD as well as ALS. The lack of regulatory approval to date is primarily due to insufficient evidence demonstrating consistent clinical efficacy and disease-modifying effects in completed trials [17]. The results from these larger trials over an increased duration will be helpful to more accurately determine the functional and modulatory effects of pridopidine on these disease processes.

## 3. Pridopidine Pharmacology and Mechanisms of Action

Exploring pridopidine further helps us understand the mechanism by which it supports neuronal survival. In this regard, pridopidine acts as a neuromodulating agent that stabilizes neuronal function by facilitating signaling and enhancing cellular resistance instead of strongly stimulating dopamine receptors [15,36]. It does not act like traditional dopamine agonists or antagonists. Rather, it balances signaling between both insufficient and excessive conditions [37]. This stabilizing effect is central to its therapeutic profile. Pridopidine-mediated mechanisms target multiple molecular receptors within the nervous system, rather than relying on a single pathway like more traditional dopamine-related pharmaceuticals [15,38,39]. This mechanism allows it to adapt to different pathological conditions and thus may contribute to more consistent therapeutic effects across patients with varying disease severities. Understanding this distinct mechanism requires exploring this drug’s pharmacokinetics.

Pridopidine is effectively absorbed after oral administration, reaching its peak plasma concentration within a few hours [18]. It crosses the blood–brain barrier effectively, allowing it to act within the CNS [16]. This pharmaceutical undergoes hepatic metabolism by cytochrome P450 enzymes found in the liver and has a controlled half-life that allows it to be taken once or twice daily [35,40]. Understanding this drug’s clear absorption profile reduces the variability between patients and dosing. By maintaining effective receptor engagement, we can avoid potentially adverse fluctuations in patient drug levels. In addition, pridopidine’s ability to effectively cross the blood–brain barrier ensures that therapeutic concentrations are achieved at the site of action rather than remaining in peripheral circulation [16]. These pharmacokinetic properties ensure that the brain is consistently exposed to the drug, which facilitates its dopaminergic effects.

Pridopidine interacts with dopamine D2 receptors in a unique way. It has low affinity for the dopamine D2 receptor, meaning it binds weakly compared to traditional drugs [15,36]. This allows it to modulate rather than block or overstimulate signaling. The idea of functional stabilization describes how it enhances signaling when dopamine is low and decreases it when dopamine is high [37]. This balanced modulation reduces the risk of side effects seen with alternative dopaminergic drugs that may be stronger. For example, dopamine receptor blockers with higher affinity can lead to side effects with motor control, while strong dopamine agonists can lead to various dyskinesias. Pridopidine avoids these two extremes by maintaining signaling within a set physiological range. This effect, known as “state-dependent” modulation, makes pridopidine relevant in disorders in which dopaminergic tone fluctuates over time [37,38]. However, dopaminergic activity alone does not explain its full therapeutic profile, leading to interest in other targets [15,36].

Pridopidine strongly agonizes the S1R, a protein involved in cellular regulation [15,39]. It has also been found that activation of the S1R has been linked to an improvement in the efficiency of intracellular signaling and overall protection against excitotoxicity [1,6]. This is especially important in neurodegenerative conditions, where excessive stimulation can damage neurons. By moderating these processes, pridopidine and S1R signaling play an important role in maintaining cellular homeostasis under environmental stressors [1,39].

Pridopidine supports mitochondrial function and cellular health. It assists in enhancing the efficiency of energy production [6,41]. Pridopidine also reduces oxidative stress, which can damage cellular components [6]. The drug also activates anti-apoptotic signaling pathways, leading to the prevention of programmed cell death [39,41]. These combined effects protect neurons from degeneration under various stressful factors. Mitochondrial dysfunction is a key feature of many neurodegenerative diseases, and thus, by improving mitochondrial efficiency, disease progression can be substantially slowed [6]. Also, by reducing the accumulation of ROS, pridopidine helps preserve the integrity of cellular contents, including proteins, lipids, and DNA [6]. This preservation is key for maintaining long-term cellular function in high-energy-consuming organs such as the brain. Overall, this creates a much more stable intracellular environment that supports the survival of neurons in the CNS. Pridopidine essentially provides the foundation for improved neuronal communication and adaptability [6,39,41].

**Figure 1:**
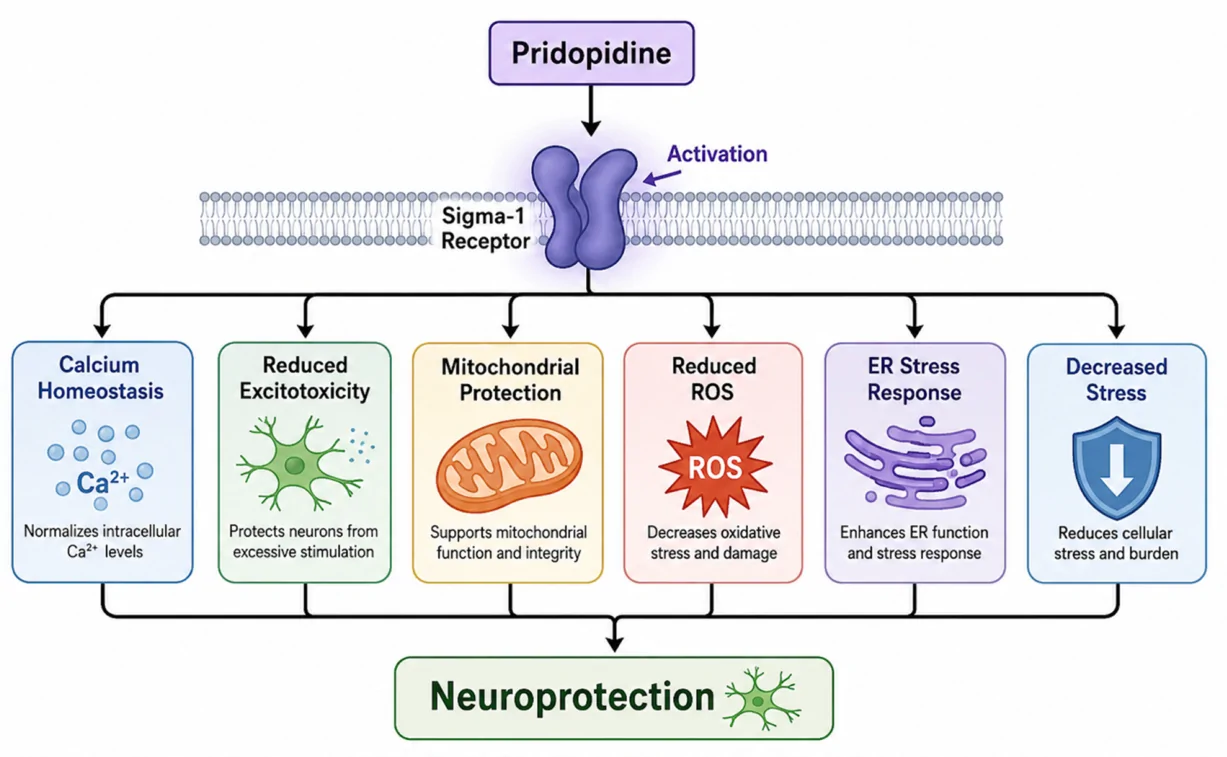
Proposed mechanisms that underlie pridopidine-mediated Sigma-1 receptor (S1R) activation and eventual neuroprotection. Activation of S1R by pridopidine enhances intracellular calcium regulation, mitochondrial function, and endoplasmic reticulum stress response pathways. It reduces excitotoxicity, reactive oxygen species-mediated oxidative stress, and cellular stress responses. These combined effects may contribute to improved neuronal resilience and survival. The schematic provides a conceptual design of the mechanisms described in the literature but does not represent a single signaling pathway. The figure was created using Open AI Chat GPT (GPT-5) with a prompt describing the proposed biological actions of pridopidine. AI generation of the figure is transparently disclosed, and the final figure was reviewed, approved, and verified for its scientific content and interpretation.

Pridopidine is also shown to enhance overall neuroplasticity and neuronal synaptic stability. Pridopidine facilitates this by increasing signaling through BDNF, a key molecule for neuronal growth and survival, and essentially improves communication between neurons and the sustainability of neuronal synapses [38,42]. This pharmaceutical also promotes healthy dendritic structure within neurons, which is essential for receiving and processing signals that cross the synapses, and for strengthening overall neural networks, improving brain function [1,42]. Pridopidine has also been found to enhance neuroplasticity, which allows the brain to adapt more effectively to injury or cellular degradation [43]. By supporting dendritic branching and synaptic density, pridopidine can help restore lost connections in damaged neural circuits [1,42]. This ability for structural adaptation is necessary for functional recovery in progressive neurological diseases and has been shown to be important in chronic neurological conditions where structural decline contributes to functional impairment. Overall, these effects of pridopidine help to stabilize and protect neuronal systems within the CNS [1,38,39,42,43]. A figure of the proposed mechanism of action of pridopidine is seen in Figure 1.

## 4. Pridopidine in Neurodegenerative Disorders

### 4.1. Huntington’s Disease (Primary Indication)

HD is an autosomal dominant, progressive neurodegenerative disorder that presents with chorea, dystonia, incoordination, cognitive decline, and behavioral difficulties. It is a trinucleotide repeat disorder caused by an expanded CAG repeat mutation in the *HTT* gene, which encodes the huntingtin protein. The inherited mutation results in the production of an elongated polyglutamine mutant huntingtin protein.

The functions of the Huntington protein are not fully understood, but its mutant variant is thought to alter gene transcription and energy production, and to dysregulate neurotransmitter metabolism, receptors, and growth factors [44]. Evidence suggests that this tail confers a toxic gain of function, but precise pathophysiological mechanisms of HD are poorly understood [45]. Previous research suggests that glutamate and dopamine neurotransmission are affected by HD. The mechanism by which dopamine modulates glutamate-induced excitation in the basal ganglia and cortex might be disrupted and can be targeted for therapy. Standard treatments for HD include antidopaminergic medications (ADM) such as vesicular monoamine transporter 2 inhibitors (VMAT2) for chorea and antipsychotics (neuroleptics) for reduction in involuntary movements and behavioral symptoms [46].

Dopamine stabilizers, such as pridopidine, act at dopamine D2 Receptors, resulting in state-dependent behavioral effects. These new dopamine–ligand compounds are thought to stabilize dysregulated psychomotor functions by modulating hyper- or hypoactive functioning in brain areas receiving dopamine input [47,48]. According to the hypotheses of the developer of pridopidine, Arvid Carlsson, pridopidine antagonizes the binding of other ligands to dopamine-D2 receptors in a manner similar to haloperidol, but with lower potency and a more restrictive receptor–binding profile [47,48]. Pridopidine preferentially blocks dopamine neurotransmission at extra-synaptic sites. This antagonism of extrasynaptic receptors by pridopidine could improve psychosis and abnormal involuntary movements from abnormal dopamine transmission in HD [47,48]. By targeting S1R, pridopidine modulates multiple pathways in HD and other neurodegenerative diseases [47,48].

#### 4.1.1. Clinical Trial Evidence

Phase II/III trials (e.g., PRIDE-HD). Functional capacity endpoints.

Among randomized controlled trials (RCTs) conducted to determine the benefits of pridopidine in patients with HD, results have been mixed. These studies used multiple measures of functional capacity endpoints, including Unified Huntington’s Disease Rating Scale (UHDRS) total motor score (TMS), MMS, and TFC. Whereas TFC measured functional independence and daily living capacity, TMS was used to assess the severity of physical motor symptoms, with a distinction between involuntary (TMS) and voluntary (MMS) movements [2,49].

#### 4.1.2. Effects on Motor Symptoms

##### Limited to Modest Improvements

Multiple RCTs have been conducted with varying functional capacity endpoints for cognitive function and motor movement. Among those focusing on motor movements, meta-analyses across four RCTs involving 1119 patients showed significantly higher UHDRS MMS in the treatment groups, with no difference in UHDRS-TMS [48,50].

In terms of efficacy, pridopidine had a clear effect on voluntary movement symptoms, with results of meta-analyses consistently showing lower MMS scores than placebo groups. The TMS scores, which included involuntary movements and eye movements, were insignificant, showing no differences between placebo and pridopidine outside of the 90 mg subgroups.

Further analysis showed improved TMS and MMS scores with dosages greater than 90 mg/day [51].

#### 4.1.3. Functional and Cognitive Outcomes

##### Preservation of Daily Function: Potential Disease-Modifying Signals

While some studies focused on UHDRS scores related to motor movements, others focused on TFC. In PRIDE-HD, a phase 2 RCT with multiple dosages tested, pridopidine showed no significant improvement in TFC at 26 weeks at any dosage, but did show improvement in only groups taking 45 mg twice daily at 52 weeks [51]. Functional capacity in other studies, such as PROOF-HD, showed no significant slowing of disease progression, measuring TFC over a 15-month treatment period [17,27]. In the Open-HART RCT, both TMS and TFC were measured over 60 months, showing less decline than in historical placebo trials [52]. Other studies have focused on disease-modifying signals and have demonstrated changes in cerebral metabolism in regions relevant to HD when treated with pridopidine [44]. Analysis of HD models in vitro and mice has shown promising effects regarding neuroprotection and disease modification. These include increases in BDNF, reduction in mutated *HTT* levels, and S1R binding and modulation [1,53]. These additional functions are other areas that need further research before the efficacy of pridopidine can be determined.

#### 4.1.4. Mechanistic Interpretation

##### Sigma-1–Mediated Neuroprotection: Synaptic Stabilization

The mechanism of pridopidine’s treatment of HD includes both its antagonistic effects on the D2 receptor as well as agonistic effects of S1R [54]. Through its antagonistic effects, competitive binding with D2R can attenuate the suppressive effects on dopamine transport and release from vesicles and increase GABA output via indirect pathways, resulting in decreased involuntary movements. Through its agonistic effects, binding to S1R can exert neuroprotective effects by increasing BDNF expression and regulating PI3/AKT signaling [54]. While the molecular mechanism of pridopidine is well-researched, the efficacy for use in HD is still undetermined.

### 4.2. Pridopidine Modulation of Motor Circuitry in Amyotrophic Lateral Sclerosis

Pridopidine has been shown to modulate the S1R pathway as an agonist, and once bound by pridopidine, S1R is activated and begins operating as a chaperone protein in a neuroprotective state [44,45]. In SOD1 G93A transgenic mouse models, there was evidence to suggest that pridopidine had a protective effect on motor neurons and delayed disease progression via S1R and many more downstream pathways that restored axonal transport and/or optimized lipid metabolism [46]. However, the HEALEY ALS Platform trial could not reproduce these findings in humans, as they found no statistical significance in disease progression between pridopidine intervention groups and placebo groups over a 24-week trial [44]. There are promising results from sub-analyses of the HEALEY ALS Platform trial that point toward beneficial outcomes of pridopidine intervention in early stages of ALS [47]. Altogether, current reports of pridopidine do not have sufficient evidence to claim that the drug is capable of positively affecting disease trajectory but can display targeted effects on motor circuitry through its relationship with S1R-mediated neuroprotection.

#### Amyotrophic Lateral Sclerosis

ALS, otherwise known as Lou Gehrig’s disease, is a neurodegenerative condition that affects the upper and lower motor neurons, resulting in spastic and flaccid paralysis throughout the body [55]. Upper motor neuron (UMN) paralysis affects the lower extremities and is related to loss of the lateral corticospinal tract and brainstem motor nuclei, presenting with hyperreflexia, spasticity, clonus, and a positive Babinski sign [55,56]. Lower motor neuron (LMN) paralysis affects the upper extremities and is due to loss of the anterior horn nuclei, presenting with dysphagia, weakness, fasciculations, and respiratory depression [56]. Typically, ALS has a midlife age of onset due to familial mutations within the *C9orf72* and *SOD1* genes [57]. Currently, ALS remains incurable and has a poor prognosis for most patients, resulting in death due to respiratory depression 2 to 5 years after diagnosis [57].

The main pathophysiology studied within ALS is the overexcitability of neurons leading to neuronal death. This mechanism is mainly due to EAA2 transporter dysfunction of astrocytes, which normally removes excess glutamate from the synaptic cleft [58]. The exact mechanism of ALS is multifactorial and complex; however, the combination of UMN and LMN paralysis is believed to result from hyperexcitability and has been attributed to a phenomenon known as the “dying forward” hypothesis [59]. This hypothesis states that hyperexcitability (due to glutamate excitotoxicity) and TAR DNA-binding protein 43 (TDP-43) accumulation within the cytoplasm of corticofugal projection neurons depict a clear mechanism of ALS descending from the cortex to the spinal cord [59]. Additionally, TDP-43 accumulation links ALS with frontotemporal dementia (FTD), which allows for ALS to also be characterized as a degenerative brain disease, and not just a spinal cord syndrome [59,60].

This specific pathophysiology is significant because most therapeutic techniques, such as Riluzole, focus on decreasing glutamate release from neurons, inactivating Na+ channels, and inhibiting NMDA receptors in order to decrease excitation within the CNS [61]. However, because ALS is multifactorial, these mechanisms only play a neuroprotective role within ALS and do not treat the underlying condition [61]. Consequently, other therapies such as pridopidine are being studied in order to determine a cure for this condition.

Pridopidine is an S1R originally intended as a treatment for HD [17]. Neurodegenerative diseases such as ALS disrupt the MAM due to the accumulation of TDP-43, which leads to cellular stress and neuronal damage [1,62]. Pridopidine agonism is able to restore this disruption by increasing the number of connections between the ER and mitochondria and preserving the stability of the MAM [1]. This maintains calcium equilibrium and prevents excitotoxicity within ALS. S1R agonists, such as pridopidine, are novel therapeutics that are unfortunately still found to be neuroprotective and not curative [1,43,62]. However, further research within the HEALEY ALS Platform trial depicts pridopidine’s therapeutic efficacy.

The HEALEY ALS Platform Trial, conducted from January 2021 to July 2022, was an evaluation of multi-drug efficacy on patient prognosis, consisting of a double blind, randomized, and multicenter study [35]. Specifically in the context of pridopidine, 163 patients were randomly given pridopidine or a placebo, and an additional 122 patients receiving placebo from other drug-regimen trials were also included [35]. The trial concluded that of all patients taking pridopidine or the placebo, there was no significant decrease in ALS progression and survival decline [35]. However, in patients who had experienced their first symptoms within 18 months of beginning the treatment regimen, there were neuroprotective factors that lowered rapid disease progression early on [35]. Using the ALS functional rating scale-revised within this subgroup (<18 months onset), it was determined by the HEALEY ALS Platform trial that patients on pridopidine displayed a 32% slowing of ALS progression, a 62% decrease in respiratory decline, an 88% reduction in dyspnea, and an increase in speech by preserving corticobulbar function [35].

Unfortunately, slowing ALS progression through neuroprotective drugs is the only treatment option currently available to patients [63]. Many novel therapeutics such as riluzole and pridopidine have different mechanisms of action but ultimately lead to the same circumstances. Forming curative therapies for ALS remains a challenge due to its complex and multifactorial nature [64]. The clinical evidence for pridopidine in ALS and HD is summarized in Table 1.

## 5. Safety Concerns and Clinical Limitations

Pridopidine has shown a consistent and favorable overall tolerability and safety outcomes in multiple clinical trials involving individuals with neurodegenerative disorders. The drug has been studied most extensively in HD, with additional data from trials in ALS. Early in its development, pridopidine was characterized as a “dopaminergic stabilizer.” More recent mechanistic and receptor occupancy studies have identified S1R agonism as its primary pharmacologic mechanism at clinically relevant doses [29,47]. This shift matters clinically because S1R modulation is linked to neuroprotective effects while avoiding many of the severe adverse reactions associated with traditional dopaminergic medications and receptor antagonists [37,65].

In large HD trials such as the PRIDE-HD phase 2 study, pridopidine demonstrated good tolerance, and the adverse event rates were comparable to the placebo [19]. The most frequently reported side effects were headache, dizziness, insomnia, fatigue, nausea, and gastrointestinal discomfort [19]. These adverse effects were generally ranging from mild to moderate in their severity and rarely resulted in treatment discontinuation. Long-term extension studies with open-label settings, some with follow-up extending to 6.5 years, have confirmed sustained tolerability without evidence of cumulative toxicity or progressive adverse neurologic effects [66]. Importantly, pridopidine does not appear to cause substantial sedation, extrapyramidal symptoms, or severe cognitive impairment effects frequently encountered with dopamine receptor antagonists and certain other centrally acting neuropsychiatric agents [36,47]. This profile is particularly advantageous for patients with neurodegenerative diseases, who often already contend with frailty, gait instability, and cognitive impairment and are therefore more vulnerable to medication-related harm [67].

Although pridopidine demonstrates favorable overall tolerability, several CNS-related adverse effects have been identified across different clinical trials. These are thought to arise from modulation of dopaminergic neurotransmission and S1R activity within central neural circuits [47,65]. In HD trials, a subset of patients experienced transient worsening of choreiform movements or motor hyperactivity, particularly during dose titration; these effects were generally dose-dependent and did not tend to persist with continued treatment [19,27]. Psychiatric symptoms such as confusion and hallucinations have also been noted, primarily among patients with advanced disease or underlying psychiatric comorbidities [67]. Reassuringly, the incidence of severe neuropsychiatric complications remains substantially lower than that reported with traditional dopaminergic therapies [36]. Recent phase 3 data from the PROOF-HD trial confirmed that rates of psychiatric adverse events and suicidality with pridopidine were consistent with the elevated background risk inherent to HD and comparable to placebo [17].

These mechanisms have generated interest in pridopidine not only as a symptomatic therapy but also as a potential disease-modifying agent. There has been no major evidence of direct neurotoxicity or accelerated neurodegeneration in current human trials [17,19]. The principal caveat is that most controlled clinical trials have been relatively short in duration (typically ≤78 weeks), although accumulating long-term open-label extension study data are reassuring regarding neurological safety [17,66].

Cardiovascular safety has received particular attention with pridopidine. Early dose-ranging studies and trial protocols raised the possibility of QT interval prolongation at higher doses and therefore incorporated extensive electrocardiographic monitoring [19]. Mild QTc changes have occasionally been observed, but clinically significant arrhythmias and major cardiovascular events have remained uncommon [19,68]. A dedicated thorough QTc study has established that at the therapeutic dose of 45 mg twice daily, pridopidine does not result in significant QTc prolongation clinically or increase arrhythmic risk [17,68]. Relative to many other CNS-active agents, pridopidine appears to have a more favorable cardiovascular profile. Low potency first-generation antipsychotics, certain second-generation agents (notably clozapine and quetiapine), and some dopamine agonists (such as pramipexole and bromocriptine) have been associated with orthostatic hypotension or autonomic dysfunction; effects that have not been prominent with pridopidine [19]. This distinction is particularly relevant in elderly patients who often have baseline cardiovascular disease and autonomic instability [67]. Nonetheless, caution remains warranted in patients with preexisting cardiac disease, electrolyte abnormalities, or concurrent use of other QT-prolonging medications [18]. In such populations, periodic electrocardiographic monitoring may be appropriate, especially when higher doses are considered or when polypharmacy is unavoidable [18].

Polypharmacy is highly prevalent among patients with neurodegenerative diseases, creating substantial potential for clinically relevant drug interactions. Patients with HD, Parkinson’s disease, and ALS commonly receive multiple medications, including antidepressants, antipsychotics, anticonvulsants, dopaminergic agents, benzodiazepines, and cognitive enhancers [18,67]. Pridopidine is primarily metabolized through hepatic cytochrome P450 pathways, particularly CYP2D6, and functions as a CYP2D6 auto-inhibitor [16,69]. Concomitant use of strong CYP2D6 inhibitors may increase plasma concentrations of pridopidine, potentially enhancing adverse effects, although major interaction-related safety signals have not emerged in clinical trials to date [69]. Coadministration of other CNS-active drugs may theoretically increase the risk for additive CNS side effects [47]. A notable limitation in assessing and characterizing the safety profile of pridopidine is the substantial heterogeneity across trials, including differences in duration, dosing regimens, subject disease severity, and methods of quantifying outcomes [17,19,70,71]. This is further complicated by the marked biological and clinical variability inherent to neurodegenerative diseases [70].

Overall, while pridopidine demonstrates favorable tolerability and mechanistic promise for neuroprotection, its modest effect sizes and inconsistent trial outcomes in some studies underscore the need for larger, longer-duration, and more standardized clinical studies to better define its therapeutic role in neurodegenerative disease management [17,70,71].

## 6. Mechanistic Integration: Sigma-1, Mitochondria, and Neuroplasticity

Emerging evidence suggests that the neuroprotective effects of pridopidine are best understood not as modulation of a single downstream pathway, but as coordinated regulation of interconnected cellular stress and neuroplasticity networks centered around the S1R. Across HD, ALS, PD, AD and related neurodegenerative models, S1R activation has been linked to stabilization of MAMs, restoration of calcium homeostasis, preservation of mitochondrial bioenergetics, reduction in oxidative stress and enhancement of BDNF-mediated synaptic plasticity [72,73]. Together, these findings support a mechanistic framework in which S1R activation couples mitochondrial resilience to neuroplasticity and circuit maintenance.

### 6.1. Sigma-1 Receptor as a Cellular Hub

The S1R is increasingly recognized as a central regulator of ER-mitochondrial communication and cellular stress adaptation. Localized predominantly at MAMs, S1R appears positioned to coordinate calcium signaling, ER proteostasis, mitochondrial function, and downstream survival pathways under neurodegenerative stress conditions.

Weng et al. described S1R as a key chaperone localized at MAMs, where it regulates intracellular Ca^2+^ signaling and maintains structural coupling between the ER and mitochondria [72]. Under stress conditions, S1R dissociates from Binding Immunoglobulin Protein (BiP) and stabilizes IP3R3, thereby facilitating controlled calcium transfer from the ER to mitochondria and supporting ATP production [72]. Similarly, Couly et al. reviewed evidence that S1R stabilizes the IP3R3-GRP75-VDAC1 complex at the MAM, reinforcing ER-mitochondrial calcium signaling and maintaining MAM integrity across neurodegenerative conditions [73].

Beyond calcium transfer, S1R also appears to regulate broader calcium homeostasis networks. Shi et al. summarized evidence that S1R modulates NMDA receptors, voltage-gated calcium channels, and store-operated calcium entry pathways, supporting its role as a broader regulator of neuronal calcium signaling [74]. Consistent with this, Ryskamp et al. demonstrated that pridopidine lowered ER calcium levels and enhanced neuronal store-operated calcium entry (nSOC) in PS1-KI Alzheimer’s disease models, whereas S1R knockdown abolished these synaptoprotective effects [75]. Together, these findings suggest that S1R may function as a dynamic calcium-sensitive signaling hub linking ER physiology to mitochondrial and synaptic homeostasis.

In parallel with calcium regulation, several studies support a role for S1R in maintaining ER proteostasis during neurodegenerative stress. Shenkman et al. demonstrated that pridopidine reduced mutant huntingtin-induced ER stress at low nanomolar concentrations and lowered markers across all three UPR pathways, with the strongest effects observed in the PERK branch [76]. Importantly, CRISPR-mediated deletion of S1R abolished these effects, confirming receptor dependence [76]. The authors further showed that mutant huntingtin disrupted S1R colocalization with the ER chaperone BiP, whereas pridopidine restored this interaction toward normal levels, supporting a role for S1R in preserving ER homeostasis under pathological conditions [76]. Couly et al. similarly reviewed evidence that SIGMAR1 stabilizes IRE1 signaling during ER stress, while loss or mutation of SIGMAR1 increased ER stress and apoptosis in ALS-related cellular models [73].

The importance of S1R as the principal mediator of pridopidine’s effects is further strengthened by genetic and pharmacologic studies. Eddings et al. demonstrated that pridopidine protected mutant huntingtin-transfected neurons and HD patient-derived iPSCs with mid-nanomolar potency, whereas S1R antagonism with NE-100 or genetic deletion of S1R abolished neuroprotection. In contrast, TrkB inhibition did not eliminate protection, suggesting that S1R signaling may act upstream of, or parallel to, canonical neurotrophin pathways [38]. Naia et al. further showed that pridopidine restored disrupted ER-mitochondrial contact sites and improved co-localization of IP3R and S1R with mitochondria in YAC128 HD neurons, directly linking S1R activation to preservation of MAM integrity [6].

#### Pridopidine in Comparison with Other Sigma-1 Receptor-Targeting Therapeutics

Pridopidine, a selective S1R agonist with D2 receptor stabilizing activity, shares common characteristics with other S1R agonists such as ANAVEX2-73, SA4503, and T-817MA, primarily through their action on S1R activation and their ability to modulate cellular pathways involved in neuronal survival, stress responses, and their use in multiple neurological disease models [77]. However, these compounds differ in their pharmacological profiles, disease indications, and clinical outcomes.

Pridopidine has primarily been investigated for HD and ALS, while ANAVEX2-73 (Blarcamesine) shows neuroprotective activity in Alzheimer’s models, reversing learning deficits, and was also found to regulate autophagy and proteostasis [1,77]. SA4503 (Cutamesine) demonstrated antidepressant like effects in animal models and improved functional recovery after stroke; however, its trial data showed no significant improvement in functional endpoints. Additionally, T-817MA (Edonerpic maleate) reduced amyloid beta-induced neurotoxicity; however, phase 2 Alzheimer’s disease trials did not show efficacy at maximal doses [77]. Overall, the S1R targeting compounds demonstrate neuroprotective potential across several neurological disease models, although clinical translation has remained variable.

### 6.2. Mitochondrial Function in Neurodegeneration

Mitochondrial dysfunction and oxidative stress are early and intersecting features across multiple neurodegenerative disorders. Bioenergetic deficits, impaired oxidative phosphorylation, ROS accumulation, disrupted mitochondrial dynamics, and defective mitophagy contribute to progressive neuronal vulnerability and degeneration. Because S1R is positioned at the ER-mitochondrial interface, it is uniquely situated to regulate these interconnected pathways.

Weng et al. demonstrated that loss of S1R function impaired mitochondrial ATP production, altered mitochondrial membrane potential, and increased susceptibility to apoptosis during ER stress [72]. S1R deficiency was also associated with fragmented mitochondrial morphology in neuronal models, supporting a direct role for S1R in preserving mitochondrial integrity and neuronal bioenergetics [72]. Couly et al. similarly emphasized that MAM integrity is critical for ATP production, ROS regulation, autophagy and mitophagy, all of which are disrupted in neurodegenerative disease states [73].

Shi et al. further linked S1R signaling to preservation of mitochondrial membrane potential, reduced cytochrome-c release, attenuation of oxidative stress, and activation of antioxidant pathways including Nrf2 signaling [74]. These mechanisms provide a biologically plausible explanation for how S1R activation may buffer neurons against chronic metabolic and oxidative stress.

Direct evidence for pridopidine-mediated mitochondrial rescue was provided by Naia et al. using HD cellular and animal models. In YAC128 neurons, HD neural stem cells and HD lymphoblasts, pridopidine improved mitochondrial respiration, increased mitochondrial elongation and motility, reduced ROS accumulation, and restored antioxidant responses [6]. Importantly, S1R knockdown abolished these protective effects, demonstrating receptor dependence [6]. Early treatment of YAC128 mice also improved motor coordination and reduced mitochondrial ROS through normalization of mitochondrial complex activity, suggesting that mitochondrial rescue may contribute to delayed symptom progression in vivo [6].

Evidence for S1R-mediated mitochondrial protection also extends beyond classical neurodegenerative disease paradigms. Geva et al. demonstrated that pridopidine preserved retinal ganglion cell survival and rescued mitochondrial dysfunction in experimental glaucoma models through S1R activation, supporting a broader role for S1R signaling in neuronal bioenergetic resilience [78].

Importantly, these mitochondrial effects are unlikely to function in isolation. Neuronal energy metabolism, calcium buffering, oxidative stress regulation, and synaptic signaling are tightly interconnected processes. Preservation of mitochondrial integrity may therefore represent an upstream requirement for sustained neuroplasticity and circuit stability under chronic neurodegenerative stress. Taken together, these findings support a model in which S1R activation stabilizes ER-mitochondrial coupling, preserves mitochondrial energetics, reduces oxidative stress, and limits apoptosis under chronic neurodegenerative conditions.

### 6.3. Neuroplasticity and Synaptic Remodeling

Beyond mitochondrial protection, increasing evidence suggests that S1R activation also regulates neuroplasticity pathways involved in synaptic maintenance, dendritic spine stability, and circuit remodeling. BDNF-related signaling appears to represent a major component of this effect, linking cellular stress adaptation to activity-dependent synaptic preservation.

Geva et al. performed genome-wide expression profiling in the rat striatum and demonstrated that pridopidine strongly upregulated BDNF-associated pathways, with highly significant enrichment of BDNF signaling genes [37]. Importantly, the effect on BDNF secretion required intact S1R signaling, directly linking S1R activation to neuroplasticity mechanisms [37]. The study also demonstrated upregulation of AKT/PI3K, glucocorticoid receptor, and D1 receptor-associated genes, several of which are downregulated in HD models [37]. Weng et al. further summarized evidence linking S1R signaling to dendritic spine development, axonal growth, and neuroplasticity [72]. S1R disruption resulted in abnormal dendritic spine morphology, whereas pridopidine increased BDNF and DARPP-32 expression and reduced mutant huntingtin aggregates through S1R-dependent mechanisms [72]. Couly et al. similarly reviewed evidence that S1R activation enhances BDNF maturation, secretion, and TrkB signaling through ERK and PI3K pathways [73]. Together, these findings suggest that S1R-mediated neuroplasticity may involve coordinated regulation of neurotrophin maturation, intracellular signaling, and structural synaptic maintenance.

Structural plasticity effects have also been demonstrated experimentally across several disease models. Estévez-Silva et al. showed that pridopidine promoted dendritic spine formation and new synapse development in primary neuronal cultures while simultaneously protecting neurons against NMDA- and H2O2-induced toxicity [79]. Compared with PRE-084, pridopidine produced more sustained activation of MAPK/ERK and PI3K/Akt signaling pathways and generated dendritic spines associated with functional synapse formation. In APP/PS1 mice, chronic pridopidine treatment improved spatial learning and memory in the Morris water maze, linking S1R-mediated structural plasticity to behavioral recovery [79]. Similarly, Ryskamp et al. demonstrated that pridopidine preserved mature mushroom-shaped dendritic spines and reversed long-term potentiation deficits in hippocampal cultures exposed to Aβ42 oligomers [75]. Rescue effects were also observed in APP-KI and PS1-KI models, while oral pridopidine treatment restored mushroom spine density in aged PS1-KI mice in vivo. Importantly, these effects required intact S1R signaling and functional nSOC pathways, directly linking calcium homeostasis to synaptic plasticity mechanisms [75].

Using a microfluidic corticostriatal “disease-on-a-chip” platform, Lenoir et al. demonstrated that pridopidine restored impaired BDNF axonal trafficking, normalized glutamatergic transmission, corrected synaptic structural markers, rescued TrkB trafficking, and restored ERK phosphorylation in HD neurons [42]. These effects were abolished by the S1R antagonist NE100, confirming receptor dependence [42]. Notably, these findings extend the effects of S1R activation beyond isolated synaptic preservation and suggest coordinated restoration of neurotrophin trafficking, presynaptic transmission, receptor dynamics, and downstream signaling within vulnerable neuronal circuits.

Additional evidence for circuit-level neurorestoration was provided by Francardo et al. in a 6-OHDA mouse model of Parkinsonism [39]. Low-dose pridopidine improved forelimb function and abolished ipsilateral rotational bias while increasing dopaminergic fiber density and upregulating BDNF, GDNF, and phosphorylated ERK1/2. Importantly, these effects were absent in S1R knockout mice, supporting a direct S1R-dependent mechanism [39].

Furthermore, some downstream effects on BDNF signaling, synaptic remodeling, and neuronal survival may reflect secondary consequences of restored mitochondrial and calcium homeostasis rather than direct transcriptional regulation by S1R itself [80]. Collectively, these findings suggest that S1R activation promotes structural and functional neuroplasticity through coordinated regulation of calcium signaling, BDNF pathways, ERK/Akt activation, and synaptic remodeling.

### 6.4. Integrated Model of Action

Taken together, current evidence supports an integrated model in which pridopidine acts through S1R to coordinate mitochondrial protection, stress adaptation, and neuroplasticity across multiple levels of neuronal organization.

At the cellular level, S1R activation stabilizes MAM integrity, regulates ER-mitochondrial calcium signaling, limits ER stress, preserves mitochondrial energetics, reduces ROS accumulation, and suppresses apoptosis pathways [6,72,73,74]. Shenkman et al. further demonstrated that pridopidine promoted sequestration of toxic mutant huntingtin oligomers into larger, less toxic aggregates while simultaneously reducing ER stress signaling, suggesting an additional role in regulation of proteostasis pathways [76].

These cellular effects appear to extend toward synaptic and circuit-level remodeling. Lenoir et al. demonstrated restoration of corticostriatal synaptic homeostasis at multiple levels simultaneously, including BDNF trafficking, glutamate release, receptor dynamics, and downstream ERK signaling [42]. Francardo et al. similarly showed that S1R activation promoted remodeling of damaged nigrostriatal pathways in Parkinsonian mice, whereas Estévez-Silva et al. demonstrated coordinated enhancement of neuronal survival and structural plasticity through ERK and Akt signaling pathways [39,79]. Ryskamp et al. further suggested that S1R-mediated regulation of calcium signaling may mechanistically bridge mitochondrial homeostasis with synaptic stability and dendritic spine maintenance [11].

Importantly, these findings collectively suggest that pridopidine does not act through a single downstream pathway. Geva et al. demonstrated broad transcriptional activation of survival and plasticity networks, while Gershoni et al. proposed that pridopidine acts through a wider S1R-mediated resilience network involving restoration of MAM integrity, calcium homeostasis, mitochondrial function, autophagy, and BDNF-dependent synaptic plasticity [1,37]. Rather than targeting isolated disease-specific pathways, S1R activation may enhance the overall capacity of neurons to adapt to chronic neurodegenerative stress.

This mitochondria-driven neuroplasticity framework is further supported by behavioral findings across disease models. Early pridopidine treatment delayed motor impairment in YAC128 mice [6], restored cognitive performance in APP/PS1 mice [79], improved motor function in Parkinsonian mice [39], and stabilized dendritic spine density in AD models [76,77]. Similar neuroprotective effects have also been reported in retinal ganglion cell degeneration and spinal cord ischemia–reperfusion injury models, where pridopidine reduced oxidative stress, inflammation, apoptosis, and demyelination while improving behavioral recovery [78,80].

Emerging clinical findings may partially align with this mechanistic framework. In an exploratory analysis of the HEALEY ALS Platform Trial, Geva et al. 2026 observed slower decline in respiratory and bulbar function among patients with definite/probable early ALS treated with pridopidine, although these findings arose from post hoc subgroup analyses and all *p*-values were nominal [20]. While hypothesis-generating, these observations raise the possibility that S1R-mediated neuroprotective effects may be most relevant during earlier and actively progressive stages of disease, before irreversible circuit-level degeneration predominates.

An additional complexity in S1R-targeted therapeutics is the presence of biphasic or bell-shaped dose–response relationships, a pharmacologic phenomenon reported with multiple S1R agonists that may contribute to variability across preclinical and clinical studies. These nonlinear effects could reflect dynamic receptor conformational states, ligand-specific signaling bias, or differences in cellular stress context [11]. Despite strong preclinical convergence, several aspects of S1R biology remain incompletely resolved. Mechanistic findings have not always been consistently replicated across experimental systems, and the relative contribution of direct S1R-mediated signaling versus secondary downstream neuroprotective effects likely varies by disease context, neuronal subtype, and disease stage. Moreover, the pleiotropic nature of S1R signaling complicates attribution of therapeutic benefit to any single mechanistic pathway. Despite a strong mechanistic rationale, the translation of S1R-targeted therapies remains challenging due to disease heterogeneity, incomplete understanding of target engagement, and limited biomarker integration in clinical trials.

## 7. Methodological Limitations in Current Research

### 7.1. Lack of Disease-Modifying Endpoints

One of the main limitations in studies of Pridopidine is the lack of reliable disease-modifying endpoints that can clearly distinguish between symptomatic improvement and true neuroprotection. Most clinical trials in HD and ALS have relied on functional and motor-based scales such as the UHDRS and ALSFRS-R [19,20,28,51]. While these tools are clinically useful and widely accepted, they are not especially sensitive to subtle or early changes in disease progression [81]. As a result, they may miss smaller but potentially meaningful biological effects of treatment.

Most clinical trials have not incorporated strong biomarkers to confirm target engagement or track neurodegeneration (for example, neurofilament light chain or imaging-based measures) [82]. This creates a gap between what the drug is expected to do biologically and what is actually being measured in trials. Because of this, interpreting negative trial results becomes difficult. A lack of observed clinical benefit could mean the drug is ineffective, but it could also reflect that the endpoints being used are simply not sensitive enough. Additionally, the focus on symptomatic outcomes may mask potential disease-modifying effects. Some post hoc analyses, such as those from PRIDE-HD, have hinted at benefits in certain subgroups, but without endpoints that directly assess progression (like rate of decline or time-to-event outcomes), these findings are hard to draw firm conclusions from [19,54]. Overall, the absence of standardized, biomarker-informed endpoints remains a significant barrier to understanding whether pridopidine truly alters disease course.

### 7.2. Trial Design Variability

Another issue that comes up repeatedly in the literature is the degree of variability in trial design. Studies of pridopidine differ quite a bit in terms of endpoints, dosing strategies, inclusion criteria, and statistical methods, which makes it difficult to directly compare results across trials [83,84]. For example, in HD, different trials have used TMS, mMS, or composite functional measures as their primary endpoint [17,19].

Each of these captures a slightly different aspect of disease, so it is not surprising that results are inconsistent. Similarly, dose-ranging studies have not demonstrated a clear or predictable dose–response relationship. In some cases, intermediate doses appear more effective than higher ones, which raises questions about receptor saturation or possible off-target effects [17]. Patient populations also vary across studies. Differences in disease stage, genetic factors, and baseline functional status can all influence outcomes and may contribute to variability in results [85]. Furthermore, statistical approaches are not always consistent.

While some trials stick to primary analyses, others place more emphasis on exploratory or subgroup findings, which increases the risk of false-positive results [86]. In ALS research, the use of platform trials adds another layer of complexity. Shared placebo groups and adaptive designs can improve efficiency, but they can also make interpretation more challenging, particularly when trying to isolate the effect of a single intervention [20]. This level of heterogeneity makes it hard to determine whether differences in outcomes are due to the drug itself or simply differences in how the studies were conducted. Greater standardization in trial design would likely improve both reproducibility and overall interpretability.

### 7.3. Small Sample Sizes and Limited Duration

Many of the existing trials on pridopidine are also limited by relatively small sample sizes and short follow-up periods. In ALS, studies often include fewer than 300 participants and follow them for around 24 weeks [20]. HD trials are longer, but still typically only last 6 to 12 months [19,51]. Given how slowly neurodegenerative diseases progress, these timeframes may not be long enough to detect meaningful changes, especially if the treatment effect is modest [81].

This is particularly relevant for a drug like pridopidine, which is hypothesized to have neuroprotective effects that might only become apparent over longer periods [82]. Smaller sample sizes also create statistical challenges. They reduce the power to detect real differences and increase the likelihood of both false-negative and false-positive findings. Subgroup analyses can further complicate things by introducing false associations when sample sizes become even smaller [86]. Another practical issue is dropout. In progressive diseases like ALS and Huntington’s, patients may decline or withdraw from studies, and missing data can disproportionately affect smaller trials. This can introduce bias and make results harder to interpret. These limitations suggest that larger and longer trials are needed to properly evaluate pridopidine’s effects. While adaptive designs and real-world data may help address some of these challenges, they still require careful planning and standardization.

### 7.4. Translational Gaps

Finally, there is a broader issue of translation from preclinical models to human disease. In laboratory and animal studies, activation of the S1R has been associated with a range of potentially beneficial effects, including improved neuronal survival, enhanced synaptic function, and reduced cellular stress [29,87,88]. However, these findings have not consistently translated into clear clinical benefit in human trials [19,20,51].

This is not unique to pridopidine, as it reflects a more general challenge in neurodegenerative research. Preclinical models often fail to fully capture the complexity and heterogeneity of human disease [89]. As a result, treatments that appear effective in controlled experimental settings may not perform as well in clinical populations.

Another issue is the lack of well-established translational biomarkers. Without clear evidence that the drug is engaging its intended target in humans, it becomes difficult to interpret negative results [82]. For example, if a trial fails, it is unclear whether this is because the drug does not work or because it did not adequately affect the S1R in vivo.

There are also differences in how outcomes are defined across preclinical and clinical studies. Preclinical research often focuses on cellular or molecular markers, whereas clinical trials prioritize functional outcomes. This mismatch can make it harder to connect findings across different stages of research.

Addressing these translational gaps will likely require better integration of biomarkers, more representative disease models, and closer alignment between preclinical and clinical endpoints. It will remain challenging to fully assess the therapeutic potential of pridopidine without these improvements.

## 8. Future Directions

Despite five studies conducted on the effectiveness of pridopidine in treating the progression of HD, none have met their desired goals or endpoints [17,90]. The latest and largest trial to date, PROOF-HD, failed to meet endpoints partly due to the fact that these endpoints may not be sensitive enough; the trial may not have been long enough at 65 weeks, large enough at 499 participants, and they were not able to account for concomitant medications, which may have altered the results of the study [17].

It is important for future studies to address these areas where PROOF-HD failed, if any advancement is to be made in assessing pridopidine’s efficacy in delaying the symptoms of HD. A future trial should have a longer span; the 65-week span should be extended to allow for more time to collect data and meet endpoints [19]. Another shortcoming of previous trials that should be addressed moving forward is the lack of stratification of trial populations by disease stage using the Huntington’s Disease Integrated Staging System [91]. By sorting populations into more homogenous cohorts, future studies will be able to more accurately assess the efficacy of pridopidine. Additionally, increasing population size would lead to higher accuracy and precision of the trial.

Future trials should also evaluate how they can assess results using sensitive endpoints. Neuroimaging to assess caudate atrophy may be worth investigating for future trials, as other more recent trials like LEGATO-HD, which assessed a similar Huntington’s drug, adopted this measure and proved useful [92]. Past trials such as PROOF-HD found that pridopidine performed better in patients who were not taking antidopaminergic drugs for the treatment of chorea and behavioral symptoms [17]. Geva et al. presented evidence that these antidopaminergic drugs actually accelerate cognitive decline in patients with HD [93]. Future studies could exclude patients currently taking these medications or utilize stratified randomization to evenly distribute these individuals who may confound results.

Determining which biomarkers could be used to understand the mechanism of pridopidine and its efficacy may improve the chances of success in future trials. A study by Grachev et al. found that pridopidine binds strongly to S1Rs using PET [15]. Therefore, future studies should evaluate the downstream effects of sigma-1 binding and its role in neuroprotection. Additionally, neuroimaging parameters could be utilized as more sensitive measures of disease progression than traditional clinical assessments [94]. Both caudate and putamen atrophy have been shown to be an effective indication of disease progression [95]. By tracking these biomarkers, future trials could assess the downstream effects of pridopidine on neural protection across the trial populations and correlate them with treatment efficacy.

Combination therapies that integrate pridopidine with Huntington’s disease-modifying agents are a possible yet unvalidated approach that future studies could pursue. HD is caused by a CAG trinucleotide repeat disorder that results in the production of a mutant huntingtin protein [84]. Antisense oligonucleotides (ASOs) are disease-modifying agents that have shown the ability, in a study by Tabrizi et al., to reduce mHTT production by targeting huntingtin messenger RNA [96]. In a phase 3 trial of the ASO tominersen, the study was halted due to a dose-related clinical worsening of symptoms at higher doses [97]. Pridopidine, on the other hand, is thought to have potentially positive downstream neuroprotective effects through the S1R [38,93]. In theory, ASOs could target the upstream production of mHTT, while pridopidine could exert its effects via downstream neuroprotection through activation of S1Rs, potentially slowing the progression of HD.

However, this is highly theoretical, as neither of these two strategies has proven to be an effective treatment for HD in their own phase 2 or 3 trials. Though no studies have been conducted, observations of each agent’s mechanism of action suggest these agents may be mechanistically complementary, but predicting how patients will respond to treatment is difficult due to the multifactorial nature of HD [84]. Additionally, pridopidine displays a biphasic dosing effect, meaning that the drug exerts different effects based on dosing levels, adding another layer of complexity to the discussion [19]. Future studies will need to determine optimal dosing and track relevant biomarkers to determine whether these drugs could exert synergistic effects in the treatment of HD [94]. This challenge is compounded by the fact that pridopidine has now failed to meet primary endpoints across four phase 2–3 trials [17], and the ASO tominersen caused dose-dependent clinical worsening of symptoms at higher levels of exposure [97].

Pridopidine could be investigated as a potentially therapeutic agent in disorders other than HD that are associated with neurodegeneration and CNS cellular stress. Pridopidine has been investigated as a potential therapy in ALS [20]. ALS is a complex disorder that is not completely understood, but roughly 20% of familial cases are linked to mutations in the zinc-copper superoxide dismutase 1 (SOD-1) gene [4]. This mutation causes misfolding and aggregation of SOD-1 proteins on the mitochondrial membrane, leading to mitochondrial and calcium homeostasis dysfunction [98]. Through interaction with S1Rs, pridopidine modulates cellular stress and mitochondrial dysfunction [37]. In studies with SOD-1 mouse models, pridopidine reduced mutant SOD-1 aggregation and reduced neuronal cell death through S1R activation [65]. However, in phase 2/3 of the HEALEY ALS trial, it was determined that pridopidine did not impact disease progression over the 24-week span [35]. However, a post hoc subgroup analysis did indicate potential benefits in slowing the respiratory decline in the early stages of ALS [20].

Neurodegenerative disorders are another possible indication for pridopidine. S1R activation may modulate neuronal stress, support mitochondrial function, and promote cell survival in disorders such as Alzheimer’s and Parkinson’s [99]. Pridopidine’s selective activation of these receptors may provide neuroprotection in these disorders, though further studies are needed to investigate this relationship.

Pridopidine has also been identified as a potential therapeutic candidate in disorders such as Wolfram and Rett syndrome. Wolfram syndrome is a rare disorder that causes defects in mitochondrial ER calcium homeostasis [100]. Preclinical studies of Wolfram syndrome have shown that sigma-1 agonists can restore calcium homeostasis and mitochondrial function in animal models [91], suggesting that pridopidine may have similar potential. Rett syndrome is caused by a mutation of the MECP2 gene, which negatively impacts BDNF [90]. BDNF plays a key role in synaptic plasticity and in neuroprotection against excitotoxicity and oxidative stress. Interestingly, S1R agonists are thought to enhance the function of BDNF in Rett syndrome [1]. The S1R agonist blarcamesine has already demonstrated preclinical success in models of Rett syndrome [101]. Pridopidine’s potential use as a therapeutic agent in both of these cases is simply due to its agonism of S1Rs and preclinical evidence of potential benefits of targeting this receptor [1].

## 9. Conclusions

Across preclinical and early clinical studies, pridopidine has emerged as a promising S1R–mediated neuroprotective agent with effects spanning mitochondrial biology, calcium homeostasis, ER stress regulation, oxidative stress reduction, and neuroplasticity. Rather than acting through a single symptomatic pathway, accumulating evidence suggests that pridopidine engages interconnected cellular resilience networks centered around the S1R and the ER–mitochondrial interface.

Mechanistically, the strongest evidence supports a model in which S1R activation stabilizes MAM integrity, preserves ER–mitochondrial calcium signaling, supports mitochondrial bioenergetics, reduces ROS accumulation, and enhances BDNF-dependent synaptic remodeling. Experimental studies across HD, ALS, PD, and AD models consistently demonstrate improvements in mitochondrial function, dendritic spine stability, synaptic signaling, and circuit-level behavioral outcomes following pridopidine treatment [6,39,42,72,75]. Together, these findings provide a strong mechanistic rationale for continued investigation of S1R agonism as a disease-modifying strategy in neurodegeneration.

However, clinical efficacy remains evolving. In the HEALEY ALS Platform Trial, Hayden et al. found that pridopidine did not significantly slow disease progression over 24 weeks despite favorable tolerability and compelling preclinical rationale [35]. Similarly, the phase 3 PROOF-HD trial failed to meet primary and secondary endpoints in the overall Huntington’s disease population, although subgroup analyses suggested potential benefit in participants not receiving antidopaminergic medications [17]. These findings underscore the ongoing challenge of translating mechanistic neuroprotection into measurable clinical benefits in heterogeneous neurodegenerative disorders. Despite strong mechanistic rationale, translation of S1R-targeted therapies remains challenging due to disease heterogeneity, incomplete understanding of target engagement, and limited biomarker integration in clinical trials [67,102]. These limitations may partially contribute to the discrepancy between robust preclinical findings and inconsistent clinical efficacy across neurodegenerative disease populations.

Importantly, recent exploratory analyses suggest that treatment effects may become more detectable in biologically selected subgroups, particularly patients with earlier and more rapidly progressive disease stages. Geva et al. 2026 reported slower decline in respiratory and bulbar function in exploratory ALS subgroup analyses despite negative overall primary endpoints, supporting the possibility that disease stage and underlying circuit biology may influence responsiveness to S1R-targeted therapies [20].

The future of pridopidine likely depends on more mechanism-driven clinical development strategies. Future studies should assess pridopidine-mediated effects across specific genetic backgrounds to determine whether therapeutic efficacy differs among genetically defined populations. In addition, biomarker-centered clinical trials could help confirm target engagement and identify the molecular pathways through which pridopidine exerts its neuroprotective effects. Improved patient stratification, optimization of S1R-targeted dosing, longer-duration studies, and incorporation of biomarkers reflecting mitochondrial function, oxidative stress, calcium homeostasis, and target engagement may be necessary to fully evaluate therapeutic potential. As proposed by Gershoni et al., pridopidine may ultimately represent a disease-modifying approach centered on restoration of core cellular resilience pathways rather than isolated symptomatic targets [1]. If future trials can successfully link these mechanistic effects to durable clinical outcomes, S1R agonism may emerge as a broader therapeutic framework for neurodegenerative disease rather than a disease-specific intervention alone.

## Figures and Tables

**Table 1:** Clinical trial evidence summary for pridopidine.

Disease	Study	Study Design	Dose	Duration	Outcome
Huntington’s Disease (HD)	PRIDE-HD	Phase II Randomized Control Trial	Multiple doses; improvement observed in the 45 mg twice daily group	26 and 52 weeks	No significant improvement in total functional capacity (TFC) at 26 weeks. Improvement in TFC observed in the 45 mg twice daily group at 52 weeks [19]
Huntington’s Disease (HD)	PROOF-HD	Randomized Control Trial	Placebo, pridopidine 45 mg/day, or 90 mg/day	15 months	No significant slowing of disease progression based on TFC measurements [17,27]
Huntington’s Disease (HD)	Open-HART	Randomized Control Trial	Pridopidine 20 mg/day, 45 mg/day, 90 mg/day	60 months	TMS and TFC measurements showed less decline compared with historical placebo trials [28]
Amyotrophic Lateral Sclerosis (ALS)	HEALEY ALS Platform Trial	Double-blind, randomized, multicenter clinical trial	Pridopidine 45 mg twice daily vs. placebo	24 weeks	No significant decrease in ALS progression or survival decline. Subgroup analyses for patients with symptom onset within 18 months of treatment showed slower ALS progression [35]

## Data Availability

No new data were created or analyzed in this study. Data sharing is not applicable to this article.
